# The impact of the emergency medical services (EMS) automation system on patient care process and user workflow

**DOI:** 10.1186/s12911-021-01658-9

**Published:** 2021-10-25

**Authors:** Faezeh Afzali, Yunes Jahani, Fatemeh Bagheri, Reza khajouei

**Affiliations:** 1 Department of Health Information Sciences, Faculty of Management and Medical Information Sciences, Kerman University of Medical Sciences, Kerman, Iran; 2 Modeling in Health Research Center, Institute for Futures Studies in Health, Kerman University of Medical Sciences, Kerman, Iran

**Keywords:** Emergency medical services, Patient care process, User, Workflow

## Abstract

**Background:**

One of the important components of the health system is the emergency medical services (EMS) system. The EMS system was implemented at Kerman University of Medical Sciences teaching hospitals to communicate the situation of patients being transferred to the hospital by EMS and to provide facilities tailored to the patient's condition. The objective of this study was to investigate the impact of the EMS system on the patient care process and the workflow of users.

**Methods:**

The hospital information system (HIS) report was used to investigate the impact of the EMS system on the patient care process and a questionnaire was distributed among 244 participants to determine its impact on the workflow of the users. Mann–Whitney U was used to analyze HIS reports, and Chi-square was used to analyze the data collected by questionnaires.

**Results:**

The EMS system reduced the patient's stay in hospital by an average of 3 h and 45 min. It also increased the number of patients' discharge from the emergency room to 2.2% and reduced the death rate by 1.3% (*p* < 0.001). Besides, 78% of physicians, 75% of nurses and 83% of technicians stated that this system has positively influenced their workflow.

**Conclusions:**

The EMS system reduced the patient's stay in hospital and mortality, and increased the speed of patient service, readiness of users to provide patient care and the number of discharged patients. However, problems such as inappropriate technical infrastructure of the EMS system should be solved to improve patients' recovery, reduce mortality and improve user satisfaction.

## Introduction

Emergency medical services (EMS) System is one of the most important parts of the health care system [1] which plays a key role in providing pre-hospital services [2]. The first Emergency Medical Services system was created by Larry Napoli's doctor, in the German-Austrian war with France [3]. In Iran in 1972, following the collapse of Tehran's Mehrabad airport roof and a large number of people were injured, an emergency center was set up in Tehran and several other big cities [4]. Under the 1973 law American Emergency Medical Services system, EMS system is an integrated system for providing health care services in emergency situations [5]. Today, the need for integrated and organized emergency services and the provision of quality care and prevention services in the EMS system have increased. Typically, in cities, the first contact with emergency patients is provided by the EMS [3, 6]. Emergency Medical Services are essential general medical services [7] with the purpose of providing services at the right time and using available resources [8]. Trained technicians early medical emergencies attend on the patient's bedside and take the necessary initial works, then transfer the patient to the hospital. As usual, pre-hospital care starts at the patient's bedside and ends in an emergency hospital [9], but some studies say the end of it, is the discharge of a patient from the hospital [10]. The EMS with providing primary care services to patients provides continuation of treatment by the hospital. Whatever the EMS system performance is more accurate and faster, the outcomes of patients' treatment are improved, and mortality and irreversible complications are reduced [3, 6, 11–14].

When an emergency patient is transferred to the hospital health care services should be provided as quickly as possible to save the patient’s life. Before the implementation of the EMS system, emergency technicians should complete triage forms of the patients as soon as they arrived at the hospital. Lack of information on the condition of the patients before transferring the patient the hospital was a challenge. Without this information, hospital staff could not provide facilities tailored to the patient's condition before arrival, resulting in the delayed provision of healthcare services. In order to solve this problem, the Emergency Medical Services automation system was created. Today it is necessary to have access to the patient's information in order to better provide emergency services. Access to essential information at the moment of service delivery is one of the effective factors in the EMS [15]. EMS technician can increase the support for emergency services and improve patient management by providing information from the moment it is present on the patient's bedside to deliver the patient to the hospital [16–19] and improve the performance of the emergency department. Awareness of the condition of a patient during the transition to a hospital provides time to prepare the equipment and complete the required medical staff.

Various studies have shown the effectiveness of the EMS in short and long term periods [20–23]. Medical surveillance is also recognized as an important part of any EMS system, that the choice of type and route of treatment by him has a significant effect on the outcome of the patient's treatment [24]. In addition, the EMS has a positive effect on patient clinical outcomes in overall interventions [20] and can provide significant improvements in the quality of service delivery, patient care and integrated care systems [25]. As noted, if the EMS automation system cannot improve the patient's recovery and the way it serves, the goal of creating it is to provide a quantitative and qualitative improvement in patient service will not be realized. It may also affect the workflow of triage physicians and nurses and medical emergency technicians that are the main users, and leading to problems such as reducing their speed in providing patient service, misdiagnosis of person's disease and etc. As other studies have shown, if users have problems with the system, they cannot work properly with that system, and that all the goals of the system cannot be fully achieved [26, 27]. Considering the importance of the EMS in health care system and its impact on patients, many studies have been conducted in various fields such as cardiovascular disease and the EMS [28–30].Also, due to the importance of the EMS users' impact on the realization of patient care goals, studies were done also to identification of factors affecting the readiness of emergency technicians [31], the impact of work in the EMS on the family [32] and stressors in emergency technicians [33].

The EMS automation system has recently been launched at the teaching hospitals of Kerman University of Medical Sciences. So far, no study has looked at the impact of this system on all three user groups involved with it. Since the goal of this system is to provide better service to the patient, and the users involved in the EMS automation system play an essential role in providing better patient service; in this study, the impact of the emergency medical services (EMS) automation system on the patient care process and user's workflow at the three teaching hospitals of Kerman University of Medical Sciences were reviewed.

## Methods

### System and setting

**Fig. 1:**
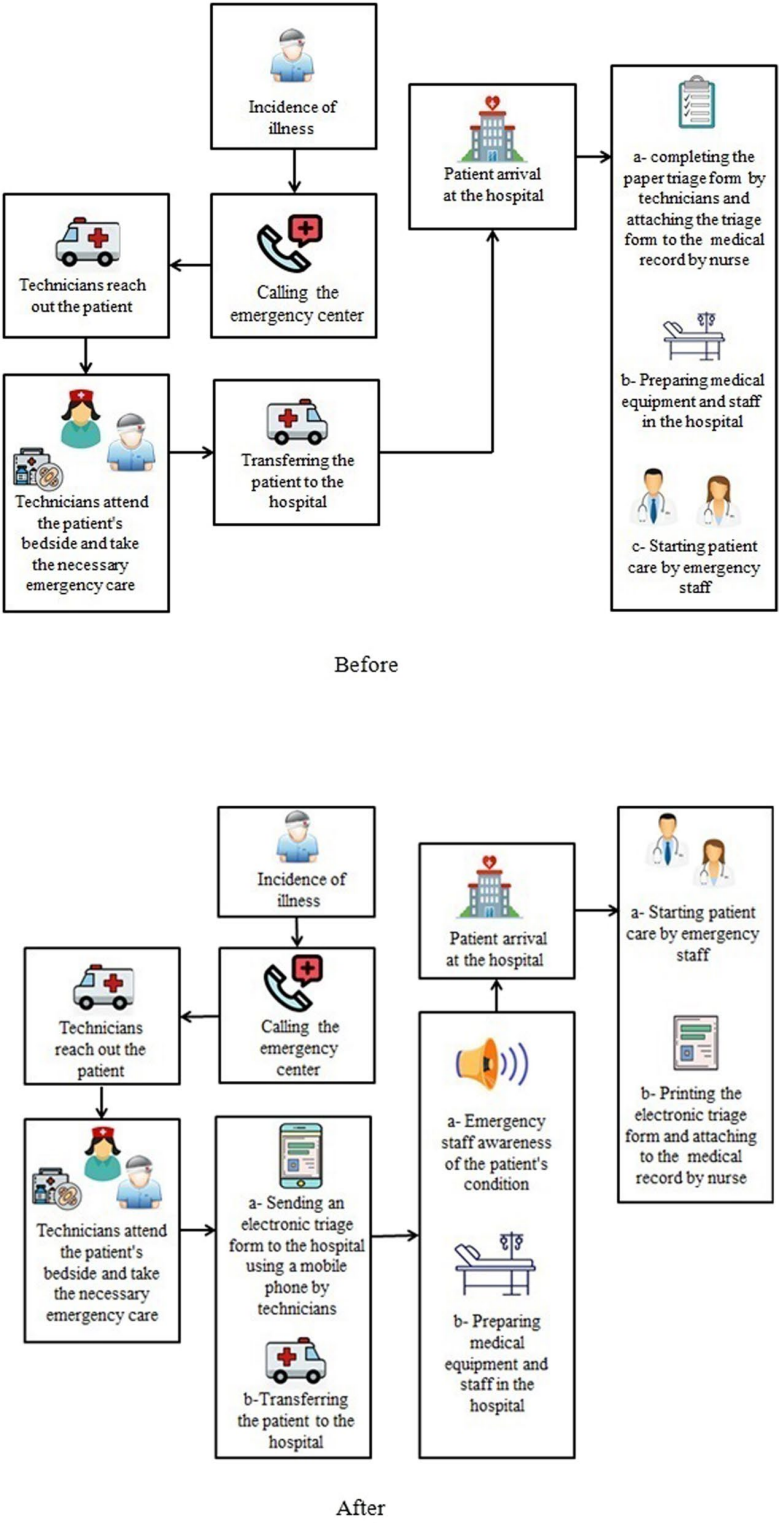
An illustration of the user workflow before and after the implementation of the EMS system

The study was carried out to evaluate the effect of the emergency medical services (EMS) automation systems on the patient care process. The secondary objective of this study was to investigate the impact of the EMS automation system on the workflow of users. The EMS automation system was implemented to provide patient information to hospital staff before patient reaching the hospital. This system was first implemented in Kerman to provide more and better patient service in August 2014, so that if the implementation of this plan is successful in Kerman, this project will be implemented throughout the country. By implementing the EMS automation system, patient data and vital signs are immediately transmitted to the physician. This project was officially conducted in Kerman University of Medical Sciences teaching hospitals in April of 2016. In the EMS automation system, emergency technicians send an electronic triage form to the hospital using a mobile phone. This electronic form that communicates the patient's condition, before the patient enters the hospital, replaces the previous paper triage form completed after the patient arrivals. For this reason, the Center of Incident Management and Medical Emergencies has provided IPhone mobile to technicians. At teaching hospitals of Kerman University of Medical Sciences, a computer is equipped with an EMS automation system and speakers for the physician and nurse awareness of the patient's condition before arriving at the hospital. If the patient's condition is very critical, the speaker connected to the system will start a siren. Therefore, emergency physicians and nurses of the triage ward can provide the necessary facilities and be prepared for delivering health care services as soon as the patient arrives at the hospital (Fig. 1).

### Study population

A total of 83 doctors and 95 nurses working in the emergency triage department of Kerman University of Medical Sciences teaching hospitals, and 66 medical emergency technicians working at Kerman Center of Incident Management and Medical Emergencies were invited to participate in the study. These technicians had at least a bachelor’s or Associate degree in emergency medicine and were trained to deliver all emergency services to patient form the incidence of illness to the time they are transferred to a hospital.

### Data collection tools and methods

This research consists of two phases:

*Phase 1* Investigation the impact of EMS automation system on patient care process

In this phase, a report from the Statistics and Information Technology Center of Kerman University of Medical Sciences teaching hospitals in order to collect the patient's identity and clinical information for comparing the length of patient stay in the hospital, the place where the patient is transferred after leaving the emergency department and the patient's condition at the time of discharge was received at six months before and after the launch of the EMS automation system.

*Phase 2* Investigation the impact of EMS automation system on user workflow

In this phase, three semi-structured questionnaires fitting the information needed to conduct research for each group of users was created by the researcher, and the content validity of the questionnaires was confirmed by two experienced faculty members in the field of medical informatics and two medical specialists of emergency medicine. Reliability of the questionnaires was evaluated and confirmed by Cronbach's alpha (physicians: α = 0.814, nurses: α = 0.815 and technicians: α = 0.824). These questionnaires consist of three parts. The first part consists of six questions for collecting demographic information such as age, sex, education, marital status, work experience and type of employment. The second part consists of 23 questions for physicians, 22 questions for nurses and 20 questions for technicians, which includes questions based on research objectives with three Likert scale (yes, somewhat, no). The third part consists of two open questions was to gather suggestions and criticisms from users regarding to the EMS automation system. A paper questionnaire in Persian was used to collect data. When filling out the questionnaires, participants were assured about confidentiality of the responses.

### Data analysis

*Phase 1* Investigation the impact of EMS automation system on patient care process

The received reports in the Excel file format were entered into the statistical analysis software, and according to the time interval of the study, they were divided into two groups, six months before the launch of the EMS automation system and six months after it. Each group includes the variables of sex, age, date and time of referral of the patient, the date and time of the patient's discharge, the place where the patient was transferred after leaving the emergency department, the patient's condition during discharge, and the length of stay of the patient in the hospital.

Since the index of Skewness and Kurtosis are very large and outside of the range of (− 1 to + 1), normalization of the data is rejected. For this reason, nonparametric tests were used to analyze the data. Frequency and percentage the place where the patient was transferred after leaving the emergency department and the patient status at the time of discharge, before and after the launch of the EMS automation system was reported, and to assess the equal distribution of these variables in two groups of patients Pearson Chi-Square test has been used. The mean, standard deviation and 95% confidence intervals of the patients' stay in the hospitals before and after setting up the EMS automation system were reported in minutes. The Mann–Whitney U test was used to measure the equal distribution of this variable in two groups of patients.

*Phase 2* Investigation the impact of EMS automation system on user workflow

In order to analyze the data of the questionnaires, the data of each group of users were entered into the statistical analysis software separately. In order to investigate the impact of the EMS automation system on the speed of patient care provision improvement, patient treatment outcome, user readiness for patient care, service delivery precision, speed diagnosis of the disease, care accuracy, time saving, the users workflow and the determination of the users problems with the system were used frequency and percentage for reporting the frequency of users responses to each question. Also, in order to measure the equal distribution of the triple responses for each question, Chi-Square test was used.

## Results

### Analysis of the impact of EMS automation system on patient care process

Table 1 show the average duration of patients' stay in the hospital, the location where the patient goes after leaving the emergency department, and the status of the discharge of patients transferred by the EMS, before and after the launch of the system. The number of patients transferred to the hospital by the EMS was six months before the launch of the EMS automation system for a total of 58,935 and 63,539 after six months. Fifty-five percent of the patients before the launch of the EMS automation system and 56% after that were woman with an average age of 39.7 and 39.4 after that, which is not significant. The results showed that the average duration of patient's stay in hospital after the launch of the EMS automation system decreased by 3 h and 45 min, which is significant. The rate of patient discharge has increased by 2.2% after the launch of the EMS automation system, which is significant. The distribution of the status during the discharge of the patient was significant before and after the implementation of the EMS automation system. What's important is a reduction of 1.3 deaths, which is significant.

**Table 1:** Patients’ length of stay, transfer location and discharge status, before and after EMS automation system

Variable			Before	After	*p*
Average duration of patients stay in the hospital (h)		Average ± (95% CI^+^)	51.77 ± (50.89, 52.64)	48.02 ± (47.29, 48.75)	< 0.0001
Where the patient is transferred	Out of the hospital	Frequency (%)	36,022(61.1)	40,206(63.3)	< 0.0001
	Transfer to another department	Frequency (%)	22,913(38.9)	23,333(36.7)	
Discharge status of patients	Recovery	Frequency (%)	42,008(71.3)	45,571(71.7)	< 0.0001
	Discharge with personal desire	Frequency (%)	8201(13.9)	7913(12.5)	
	Disgusting	Frequency (%)	814(1.4)	958(1.5)	
	Transfer	Frequency (%)	4498(7.6)	6187(9.7)	
	Follow-up	Frequency (%)	521(0.9)	652(1.0)	
	Death	Frequency (%)	2893(4.9)	2258(3.6)	

^+^*CI* confidence intervals

### Analysis the impact of EMS automation system on user workflow

In this study, out of 244 participants, 223 of them (91%) completed the questionnaire. In total, out of 83 doctors, 68 of them (82%), Out of 95 nurses, 89 of them (94%), and out of 66 emergency medical technicians, all of them (100%) completed the questionnaire. Table 2 shows the demographic characteristics of the users of the EMS automation system.

**Table 2:** Demographic characteristics of users of the EMS automation system

Variable		Doctors	Nurses	Technician
		Frequency (%)	Frequency (%)	Frequency (%)
Sex	Male	55(80.9)	22(24.7)	66(100)
	Female	13(19.1)	67(75.3)	0(0)
Age(years)	20–30	2(2.9)	34(38.2)	45(68.2)
	31–40	37(54.4)	44(49.4)	19(28.8)
	41–50	26(38.3)	10(11.3)	2(3.0)
	51–60	3(4.4)	1(1.1)	0(0)
Work experience(years)	0–5	13(19.1)	44(49.4)	31(47.0)
	6–10	37(54.4)	29(32.6)	28(42.4)
	11–15	11(16.2)	9(10.1)	6(9.1)
	16–20	6(8.8)	4(4.5)	1(1.5)
	21–25	1(1.5)	3(3.4)	0(0)
Educational status	General practitioner	63(92.6)	–	–
	Specialist	5(7.4)	–	–
	Associate degree	–	–	26(39.4)
	Bachelor's degree	–	65(73.0)	34(51.5)
	Master's degree	–	24(27.0)	5(7.6)
	Ph.D	–	0(0)	1(1.5)
Marital status	Single	23(33.8)	35(39.3)	20(30.3)
	Married	38(55.9)	49(55.1)	43(65.2)
	Separation from the partner	6(8.8)	4(4.5)	3(4.5)
	Partner's death	1(1.5)	1(1.1)	0(0)
Employment status	Permanent employment	43(63.2)	34(38.2)	8(12.1)
	Fixed term contract	25(36.7)	47(52.8)	35(53.0)
	Freelance contract	0(0)	8(9.0)	23(34.9)

### Analysis of questionnaires questions

The answer to the questionnaire questions of each group of users is shown in Table 3. The results showed that the distribution of option Yes, Some and No, are not the same.

**Table 3:** Answer to questionnaire questions by EMS automation system users

Variable		Yes frequency (%)	Some frequency (%)	No frequency (%)	*p*
1: Does the EMS automation system make you aware of the patient to be brought to the hospital sooner?	Doctors	49(72.1)	13(19.1)	6(8.8)	< 0.0001
	Nurses	81(91.0)	3(3.4)	5(5.6)	< 0.0001
	Technicians	–	–	–	–
2: Does the EMS automation system increase your readiness to provide patient care?	Doctors	43(63.2)	16(23.5)	9(13.3)	< 0.0001
	Nurses	65(73.0)	13(14.6)	11(12.4)	< 0.0001
	Technicians	–	–	–	–
3: Does the EMS automation system diagnose a patient disease faster?	Doctors	41(60.3)	16(23.5)	11(16.2)	< 0.0001
	Nurses	–	–	–	–
	Technicians	–	–	–	–
4: Does the EMS automation system increase the speed of your patient's care provision?	Doctors	47(69.1)	16(23.5)	5(7.4)	< 0.0001
	Nurses	57(64.0)	15(16.9)	17(19.1)	< 0.0001
	Technicians	49(74.2)	9(13.7)	8(12.1)	< 0.0001
5: Does the EMS automation system improve your performance for patient care?	Doctors	42(61.8)	14(20.6)	12(17.6)	< 0.0001
	Nurses	59(66.3)	13(14. 6)	17(19.1)	< 0.0001
	Technicians	–	–	–	–
6: Does the EMS automation system increase your precision to provide patient care?	Doctors	39(57.4)	17(25.0)	12(17.6)	< 0.0001
	Nurses	53(59.6)	28(31.4)	8(9.0)	< 0.0001
	Technicians	–	–	–	–
7: Does the EMS automation system make any decision about how to care for the patient beforehand?	Doctors	48(70.6)	16(23.5)	4(5.9)	< 0.0001
	Nurses	61(68.5)	19(21.4)	9(10.1)	< 0.0001
	Technicians	–	–	–	–
8: Does the EMS automation system make it easier to decide how to care for a patient?	Doctors	48(70.6)	16(23.5)	4(5.9)	< 0.0001
	Nurses	60(67.4)	19(21.4)	10(11.2)	< 0.0001
	Technicians	–	–	–	–
9: Does the EMS automation system make it possible for you to adjust your time well to provide patient care?	Doctors	39(57.4)	19(27.9)	10(14.7)	0.002
	Nurses	56(62.9)	13(14.6)	20(22.5)	< 0.0001
	Technicians	–	–	–	–
10: Does the EMS automation system reduce your stress level when providing care to the patient?	Doctors	2(2.9)	10(14.7)	56(82.4)	< 0.0001
	Nurses	2(2.2)	10(11.3)	77(86.5)	< 0.0001
	Technicians	–	–	–	–
11: Does the EMS automation system reduce your mistakes when providing care to the patient?	Doctors	46(67.6)	11(16.2)	11(16.2)	< 0.0001
	Nurses	63(70.8)	11(12.4)	15(16.8)	< 0.0001
	Technicians	–	–	–	–
12: Does the EMS automation system improve the outcome of patient care?	Doctors	46(67.7)	12(17.6)	10(14.7)	< 0.0001
	Nurses	66(74.2)	17(19.1)	6(6.7)	< 0.0001
	Technicians	38(57.6)	21(31.8)	7(10.6)	< 0.0001
13: Does the EMS automation system improve your coordination and communication with other personnel?	Doctors	44(64.7)	18(26.5)	6(8.8)	< 0.0001
	Nurses	55(61.8)	14(15.7)	20(22.5)	< 0.0001
	Technicians	48(72.7)	12(18.2)	6(9.1)	< 0.0001
14: Does the EMS automation system make sense of solidarity between the care team?	Doctors	44(64.7)	18(26.5)	6(8.8)	< 0.0001
	Nurses	54(60.7)	15(16.8)	20(22.5)	< 0.0001
	Technicians	48(72.7)	12(18.2)	6(9.1)	< 0.0001
15: Does the EMS automation system reduce your independence and freedom of action?	Doctors	2(2.9)	5(7.4)	61(89.7)	< 0.0001
	Nurses	4(4.5)	21(23.6)	64(71.9)	< 0.0001
	Technicians	3(4.5)	13(19.7)	50(75.8)	< 0.0001
16: Does the EMS automation system reduce your workload?	Doctors	1(1.5)	3(4.4)	64(94.1)	< 0.0001
	Nurses	2(2.2)	8(9.0)	79(88.8)	< 0.0001
	Technicians	5(7.6)	19(28.8)	42(63.6)	< 0.0001
17: Does the EMS automation system Worried about punishment in case of error?	Doctors	2(2.9)	7(10.3)	59(86.8)	< 0.0001
	Nurses	12(13.5)	24(27.0)	53(59.5)	< 0.0001
	Technicians	38(57.6)	19(28.8)	9(13.6)	< 0.0001
18: Dose the EMS automation system worrying about your future career if you have a problem?	Doctors	2(2.9)	7(10.3)	59(86.8)	< 0.0001
	Nurses	11(12.4)	26(29.2)	52(58.4)	< 0.0001
	Technicians	38(57.6)	19(28.8)	9(13.6)	< 0.0001
19: Does the EMS automation system make your colleagues change your behavior in case of mistakes?	Doctors	5(7.4)	10(14.7)	53(77.9)	< 0.0001
	Nurses	21(23.6)	15(16.9)	53(59.5)	< 0.0001
	Technicians	11(16.7)	13(19.7)	42(63.6)	< 0.0001
20: Does the EMS automation system affect your workflow?	Doctors	53(77.9)	13(19.2)	2(2.9)	< 0.0001
	Nurses	67(75.3)	15(16.8)	7(7.9)	< 0.0001
	Technicians	55(83.3)	3(4.6)	8(12.1)	< 0.0001
21: Does the EMS automation system increase your job security feel?	Doctors	2(2.9)	10(14.7)	56(82.4)	< 0.0001
	Nurses	6 (6.8)	14(15.7)	69(77.5)	< 0.0001
	Technicians	3(4.5)	6(9.1)	57(86.4)	< 0.0001
22: Dose the EMS automation system responsible for the results of the error only for you?	Doctors	2(2.9)	3(4.4)	63(92.7)	< 0.0001
	Nurses	12(13.5)	3(3.4)	74(83.1)	< 0.0001
	Technicians	8(12.1)	18(27.3)	40(60.6)	< 0.0001
23: Does the EMS automation system make it a legal responsibility for patient treatment?	Doctors	2(2.9)	4(5.9)	62(91.2)	< 0.0001
	Nurses	12 (13.5)	3(3.4)	74(83.1)	< 0.0001
	Technicians	–	–	–	–
24: Does the EMS automation system make your time wasting to send patient information to the hospital?	Doctors	–	–	–	–
	Nurses	–	–	–	–
	Technicians	44(66.7)	14(21.2)	8(12.1)	< 0.0001
25: Does the EMS automation system make you unnoticed by your patient?	Doctors	–	–	–	–
	Nurses	–	–	–	–
	Technicians	3(4.6)	9(13.6)	54(81.8)	< 0.0001
26: Does the EMS automation system make the patient feel overlooked by you?	Doctors	–	–	–	–
	Nurses	–	–	–	–
	Technicians	60(90.9)	4(6.1)	2(3.0)	< 0.0001
27: Does the EMS automation system make the patient feel uneasy about the patient?	Doctors	–	–	–	–
	Nurses	–	–	–	–
	Technicians	60(90.9)	4(6.1)	2(3.0)	< 0.0001
28: Does the EMS automation system disruption to your work?	Doctors	–	–	–	–
	Nurses	–	–	–	–
	Technicians	3(4.5)	12(18.2)	51(77.3)	< 0.0001
29: Dose the EMS automation system worrying about leaving your mobile phone?	Doctors	–	–	–	–
	Nurses	–	–	–	–
	Technicians	64(97.0)	1(1.5)	1(1.5)	< 0.0001
30: Does the EMS automation system make you feel satisfied during the work?	Doctors	–	–	–	–
	Nurses	–	–	–	–
	Technicians	62(94.0)	2(3.0)	2(3.0)	< 0.0001
31: Does the EMS automation system make you feel tired at the end of the mission?	Doctors	–	–	–	–
	Nurses	–	–	–	–
	Technicians	4(6.1)	14(21.2)	48(72.7)	< 0.0001

## Discussions

### Principal finding

In this study, the EMS automation system has resulted in an average reduction of 3 h and 45 min of patient's stay in hospital and 2.2% increase in patient's discharge. Also, the mortality rate has reduced by 1.3%. Besides, 78% of physicians, 75% of nurses and 83% of technicians stated that this system has positively influenced their workflow.

According to the finding of the present study, the EMS system reduced the patient's stay in hospital and increased the number of discharged patients. Reduction of patient length of stay can reduce patient and hospital treatment costs. In addition, the patient’s returns to their home earlier and reduces psychological burden of them and their family. Also, the EMS system reduced the mortality rate, since mortality rate is one of the most important health indicators, reduction in mortality is very important. The results of this study are consistent with the results of the Fares et al. [11] study, which states the performance of the EMS system is more accurate and faster, the outcomes of the patient treatment are improved and the rate of mortality and irreparable complications is reduced. The EMS system increased the speed of patient service, readiness of users to provide patient care and the number of discharged patients; and reduced the patient's stay in hospital and mortality. However, problems such as inappropriate technical infrastructure of the EMS system should be solved to improve patients' recovery, reduce mortality and improve user satisfaction.

The results of the user's questionnaire analysis showed that most physicians and nurses of the triage said that this system would make the patient condition that is supposed to be brought to the hospital sooner, thereby providing the necessary facilities for the patient, their readiness will increase to provide care. In addition, it is possible for them to diagnose a patient's disease faster. On the other hand, being aware of the patient's condition on the way to the hospital increases the speed and accuracy of their care for the patient and improves their performance to provide care to the patient. They can make decisions about how to care for the patient more easily and make their time well to provide care to the patient in advance. Their mistakes have also been reduced while providing care to the patient. The results of this study are consistent with the Yamada et al. [21] study findings that the use of a tablet to send patient information saves time for physicians and personnel involved in the EMS and enable them to share patient information. On the other hand, their coordination and relationship with other personnel has also improved and a solidarity relationship has been established between the care team, which is consistent with the results of the Langabeer et al. [28] study, which states that the transmission of the patient through the EMS increases coordination in the care system. In addition, they stated that this system does not reduce their stress when providing care to the patient, their independence and freedom of action and their workload, does not increase their job security feeling. Also, this system does not lead to possible punishment, future career and behavior change of their colleagues in the event of an error, and the responsibility for the consequences of the mistakes and legal responsibility of the patient's treatment lies with them. In general, this system affects the workflow of doctors and nurses and according to their opinion, this positive impact has been evaluated by providing a better service to the patient and improving their relationship with other staff.

The results also showed that most medical emergency technicians announced that the EMS automation system would take their time to send patient information to the hospital and, as a result, their speeds are reduced to provide initial care to the patient. On the other hand, the patients and their companions feel that the patients are neglected by the technician. In addition, their workload has increased and their workflow has been affected. They also said they were worried about leaving their mobile phone resulting in possible punishments, endangering their future career and behavior change of their colleagues in the event of an error. These concerns may be due to instability of the job status for emergency technicians, because although most doctors and nurses have permanent employment or fixed term contracts but most of the technicians have fixed term or freelance contracts. Also, doctors and nurses are supported by the hospital accreditation and quality improvement committees, while technicians have no support from the hospital. In addition, coordination and relationship with other personnel has also been improved, and a solidarity relationship has been established between the care team, which is consistent with the results of the Langabeer et al. [28] study, which states that the transmission of the patient through the EMS increases coordination in the care system. On the other hand, sending patient information to a hospital prior to the arrival of the patient improves the outcome of care provided to him, which is consistent with the results of the study by Langabeer et al. [30], which states that EMS interaction has increased patient survival. They said that this system would not reduce their independence and freedom of action, do not interfere with their work, and would not increase the feeling of tiredness at the end of their mission, but would increase their sense of satisfaction. In addition, this system does not increase their job security feel, and the responsibility for the patient's treatment is limited to them. In general, this system has affected the workflow of medical emergency technicians. Considering that this system provides better service to the patient and improves their communication with other personnel, this system has been shown to give them satisfaction during work and, ultimately, to have a positive impact.

The overall results indicate the impact of this system on the workflow of triage doctors and nurses, and medical emergency technicians. All three groups of users announced that the system still has problems, including the lack of proper infrastructure for sending and receiving patient information, such as the use of Irancell internet operator, problems with triage form items and uselessness of some of them, fear of legal responsibility while happening an error and legal lack of legality in court in the event of a patient's complaint, lack of proper feedback from the patient and his companions due to lack of people's culture, worry about missing or leaving mobile phone that lowers the quality of the mission, the small size of mobile completion of the items will disrupt the triage form and the absence of this system in nongovernmental hospitals to send information indicates that it causes a problem to send information to other hospitals and the paper triage form should be completed for them.

This study had three limitations. First, we used three-level Likert scale questions in this investigation, which may result in subjective responses and may seem simplistic. However, this was the first study evaluating the effect of the EMS in Iran. Future studies can use more robust or objective methods for this investigation. Second, in this study, we measured the effect of EMS on user’s workflow and safety of patients transporting to a hospital. Since, the EMS automation system was designed to assist in both prehospital and inter-facility transportation; the possibility of applying the findings of this study to inter-facility transportations would be limited. However, at the time of this study, the three teaching hospitals affiliated to Kerman University of Medical Sciences were the only hospitals in Iran that were equipped with the EMS. Therefore, after the implementation of this system in other hospitals in the country, future studies can be done on more centers. Third, Some essential patient-related variables for example, triage levels and patient conditions at the emergency department which should have direct impacts to the duration of patients stay in the hospital, had to be considered to evaluate the effect on patient care process and user workflow. Because these variables were not defined in the HIS of the studied hospitals, we could not consider them in our study. If these variables are defined in the HIS, future studies can also focus on the effect of these variables on patient care process and user workflow. To our knowledge, no study has looked at the impact of the EMS automation system on patient care process and the workflow of users at the same time. In addition, in this study, the influence of this system on the workflow of all involved user, is examined separately.

## Conclusions

The findings of the study showed that the EMS automation system, in general, increased the speed of patient service, the readiness of users to provide patient care, the number of patients discharged and improve the outcome of the patient's treatment. On the other hand, it has reduced the patient's stay in the hospital and the mortality rate, indicating which reflects the realization of the goal of creating the system that provides better and more service to the patient. With regard to the positive effects that the EMS automation system has on the user's workflow and patient care process, it is anticipated that if the problems are solved, the patient's recovery will be improved and their mortality will be reduced. Also, by solving the problems of users, increase their sense of satisfaction, and the goal of creating this system is to deliver better and more service to the patient will be realized.

The results of this study can be used by the officials of the Ministry of Health and Medical Education and Incident Management and Medical Emergencies Centers to plan, apply and solve the problems and improve the performance of the EMS automation system in order to improve the patient care process and reduce the negative factors affecting the workflow of triage physicians and nurses and emergency medical technicians.

It is recommended that the researchers interested in this field modify the questions of the triage form to apply it to the opinion of physicians to examine the impact of the EMS automation system on improving patient's health and reducing mortality. It also examines the effect of changing triage form questions on the workflow of users of this system.

## Data Availability

The data generated and analyzed during this study are available from the corresponding author on reasonable request.

## Abbreviations

EMS: Emergency medical services
HIS: Hospital information system
